# PageRank without hyperlinks: Reranking with PubMed related article networks for biomedical text retrieval

**DOI:** 10.1186/1471-2105-9-270

**Published:** 2008-06-06

**Authors:** Jimmy Lin

**Affiliations:** 1 National Center for Biotechnology Information, National Library of Medicine, Bethesda, Maryland, USA; 2 The iSchool, University of Maryland, College Park, Maryland, USA

## Abstract

**Background:**

Graph analysis algorithms such as PageRank and HITS have been successful in Web environments because they are able to extract important inter-document relationships from manually-created hyperlinks. We consider the application of these techniques to biomedical text retrieval. In the current PubMed^® ^search interface, a MEDLINE^® ^citation is connected to a number of related citations, which are in turn connected to other citations. Thus, a MEDLINE record represents a node in a vast content-similarity network. This article explores the hypothesis that these networks can be exploited for text retrieval, in the same manner as hyperlink graphs on the Web.

**Results:**

We conducted a number of reranking experiments using the TREC 2005 genomics track test collection in which scores extracted from PageRank and HITS analysis were combined with scores returned by an off-the-shelf retrieval engine. Experiments demonstrate that incorporating PageRank scores yields significant improvements in terms of standard ranked-retrieval metrics.

**Conclusion:**

The link structure of content-similarity networks can be exploited to improve the effectiveness of information retrieval systems. These results generalize the applicability of graph analysis algorithms to text retrieval in the biomedical domain.

## 1 Background

One of the most important innovations in information retrieval over the past decade has been the development of algorithms that exploit inter-document relationships. In most cases, documents do not exist in isolation – their environments provide an important source of evidence for ranking results with respect to a user's query. This insight is captured in algorithms such as PageRank [1] and HITS [2] (also known as "hubs and authorities"). Both have been successful in Web environments, where hyperlinks provide the inter-document relationships. The two algorithms operationalize in different ways the basic idea that a hyperlink represents an endorsement of the target page by the source author.

This article considers the application of these algorithms to a different type of graph structure for text retrieval in the biomedical domain. In the absence of manually-created hyperlinks, we argue that related article networks, or networks defined by content similarity, can be treated in the same manner as hyperlink graphs. Experiments show that incorporating evidence extracted from such networks yields statistically significant improvements in document retrieval effectiveness, as measured by standard ranked-retrieval metrics. These results are consistent with previous work and generalize the applicability of graph analysis algorithms to the biomedical domain (see Section 3.2 for discussion).

**Figure 1:**
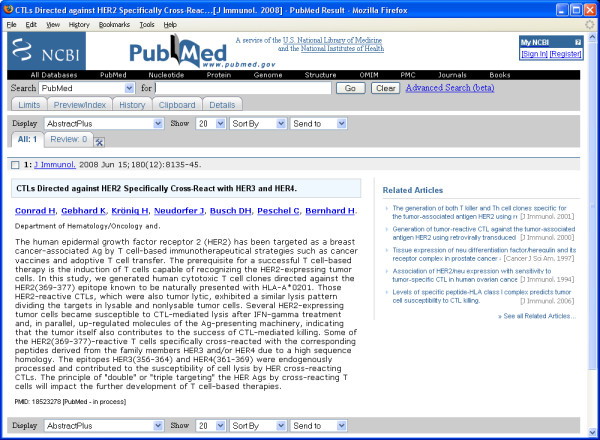
**Screenshot of PubMed showing a MEDLINE abstract.** The "Related Articles" panel on the right is populated with titles of articles that may be of interest.

The PubMed search engine provides the context for this work. Whenever the user examines an abstract in PubMed, the right panel of the browser is automatically populated with titles of articles that may also be of interest, as determined by a probabilistic content-similarity algorithm [3]; see Figure 1 for an example. In other words, each abstract view automatically triggers a related article search: the top five results are integrated into a "Related Articles" panel in the display. Note that although MEDLINE records contain only abstract text, it is not inaccurate to speak of searching for articles since PubMed provides access to the full text when available. We use "document" and "article" interchangeably in this article.

Related article search provides an effective browsing tool for PubMed users, allowing them to navigate the document collection without explicitly issuing queries. Any given MEDLINE citation is connected to a number of related articles, which are in turn connected to even more related articles, and so on. Thus, any single citation represents a node in a vast related document network defined by content-similarity links. We explore the hypothesis that these networks can be exploited for document retrieval, in the same manner as hyperlink graphs in the Web environment.

## 2 Results

### 2.1 Experimental Design

Retrieval experiments were conducted using the test collection from the TREC 2005 genomics track [4], which used a ten-year subset of MEDLINE. The test collection contains fifty information needs (called "topics") and relevance judgments, which take the form of lists of PMIDs (unique identifiers for MEDLINE citations) that were previously determined to be relevant for a given topic by human assessors. See Section 5.2 for more details. This work examines two well-known algorithms that exploit link structure to score the importance of nodes in a hyperlink graph such as the Web: PageRank [1] and HITS [2]. See Section 5.1 for a brief overview. Adapting these techniques to MEDLINE, we evaluated the impact of features extracted from related document networks in a reranking experiment.

The starting point of our experiments was a ranked list containing 40 hits, retrieved with the open-source Java-based Terrier information retrieval platform using the In_expB2 retrieval model (the *c *parameter was arbitrarily set to 1). Terrier's retrieval algorithm is based on the divergence from randomness framework [5]. Template instantiations from the genomics track topics were submitted as the queries, with no special processing.

From the ranked list for each query, we constructed several related document networks by varying the number of related document expansions for each hit in the Terrier result set. That is, for each hit, we added links to its 5, 10, 15, and 20 most similar "neighbors". Naturally, adding more related documents resulted in greater network density, which as we show has a significant impact on results. PubMed's eutil API allowed us to programmatically retrieve the related documents, which we post-processed to eliminate those not in our collection. To avoid combinatorial explosion, we did not perform second order expansions (i.e., related documents of related documents), although that is a possibility. The PageRank and HITS algorithms were then applied to these networks, using the implementation in JUNG (Java Universal Network/Graph Framework), an extensible open-source toolkit for network analysis. For PageRank, we set the random jump factor to 0.15, a value frequently suggested in the literature.

Scores extracted from the networks were combined with Terrier retrieval scores using weighted linear interpolation, controlled by the parameter *λ*, i.e., weight of *λ *to Terrier scores, weight of (1 - *λ*) to network scores. We ran three separate sets of experiments, using PageRank scores, HITS authority scores, and HITS hub scores. The output of this scoring process was a new ranking of the documents in the original Terrier ranked list. Note that related document expansions were only used to define the network over which our graph analysis algorithms (PageRank and HITS) operated – we focused only on reranking hits retrieved by Terrier.

We evaluated reranked output in terms of three standard ranked-retrieval metrics: precision at 20 documents (P20), relative mean average precision at 20 documents (MAP20), and also at 40 documents (MAP40). The cutoffs were selected to match the current PubMed interface, which displays 20 hits per page. Early precision is important in a Web search context, since users in general examine relatively few results. These metrics capture the quality of the first two result pages (since we are reranking 40 documents, P40 is not informative). Finally, note that we measure relative MAP – that is, with respect only to relevant documents contained in the original Terrier ranked list. This modification was made since we were only working with the top 40 retrieved documents; for topics with more than 40 known relevant documents, a perfect score was impossible. Different ranges on possible MAP values make meaningful cross-topic comparison and aggregation difficult. Since the test collection we used contained a mean of 95 relevant documents per topic, this was a real concern – computing relative MAP addresses these issues.

### 2.2 Retrieval Effectiveness

**Figure 2:**
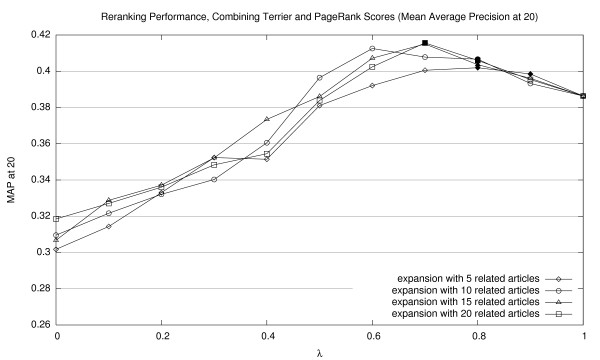
Effectiveness of interpolating Terrier retrieval scores with PageRank scores (MAP20).

**Figure 3:**
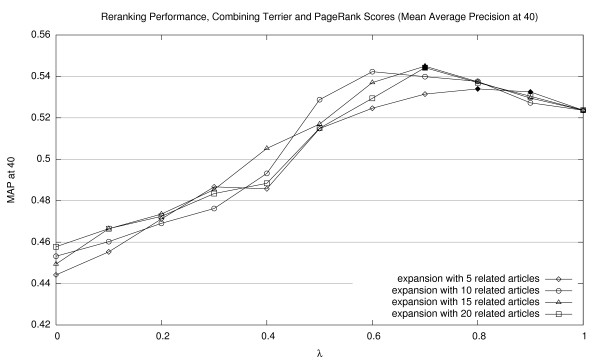
Effectiveness of interpolating Terrier retrieval scores with PageRank scores (MAP40).

**Figure 4:**
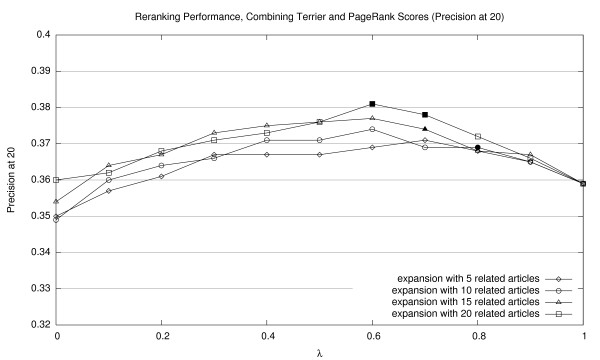
Effectiveness of interpolating Terrier retrieval scores with PageRank scores (P20).

Results of our reranking experiments combining Terrier and PageRank scores are shown in Figures 2, 3, and 4 (for MAP20, MAP40, and P20, respectively). Expansions with different numbers of related documents are shown as separate lines. The *x*-axis represents a sweep across the *λ *parameter space in tenth increments. Thus, the right edge of each line (*λ *= 1.0) represents baseline Terrier results (with no contribution from PageRank scores). For all experiments in this paper, we applied the Wilcoxon signed-rank test to assess the statistical significance of performance differences. Points that represent significant improvements over the Terrier baseline (*p *< 0.05) are denoted by solid markers. See Section 5.3 for a discussion of statistical testing and caveats on interpreting these results.

The graphs confirm that incorporating PageRank scores using our simple combination approach improves ranked retrieval effectiveness, reaching optimal scores between 0.6–0.8 in terms of *λ *values. Lower values of *λ*, representing heavier emphasis on PageRank scores, consistently results in below-baseline effectiveness. In general, we note that more related article expansions improve retrieval effectiveness. It appears that denser networks yield a better estimation of a document's importance.

**Figure 5:**
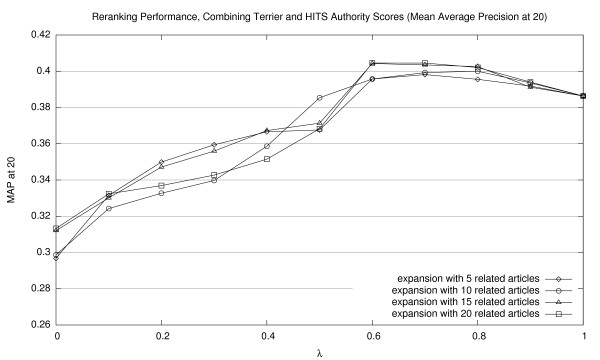
Effectiveness of interpolating Terrier retrieval scores with HITS authority scores (MAP20).

**Figure 6:**
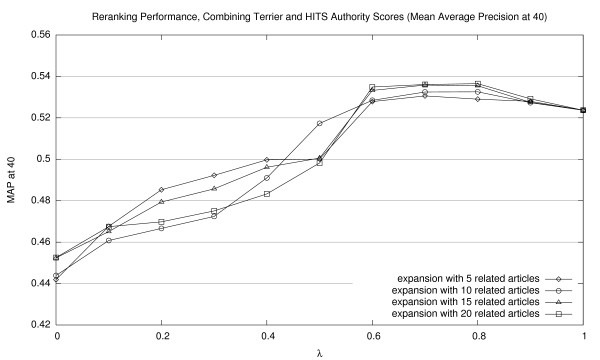
Effectiveness of interpolating Terrier retrieval scores with HITS authority scores (MAP40).

**Figure 7:**
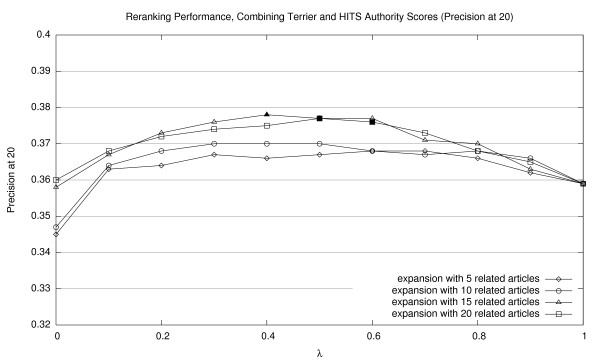
Effectiveness of interpolating Terrier retrieval scores with HITS authority scores (P20).

**Figure 8:**
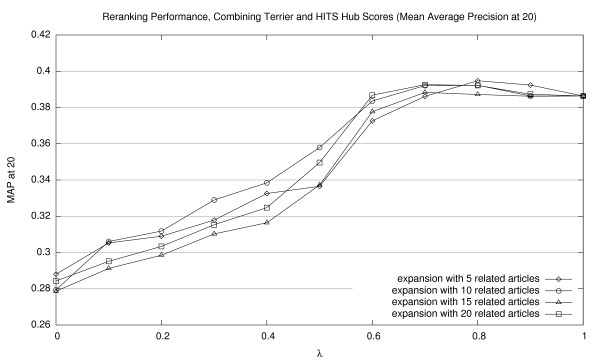
Effectiveness of interpolating Terrier retrieval scores with HITS hub scores (MAP20).

**Figure 9:**
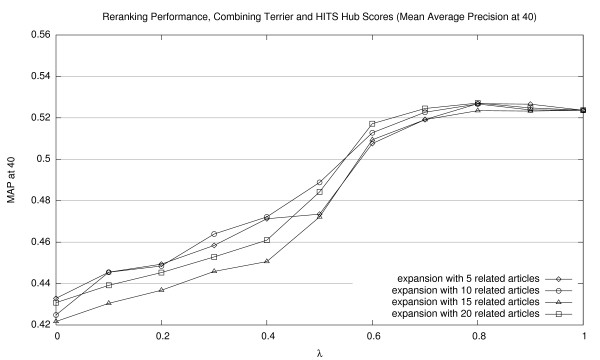
Effectiveness of interpolating Terrier retrieval scores with HITS hub scores (MAP40).

**Figure 10:**
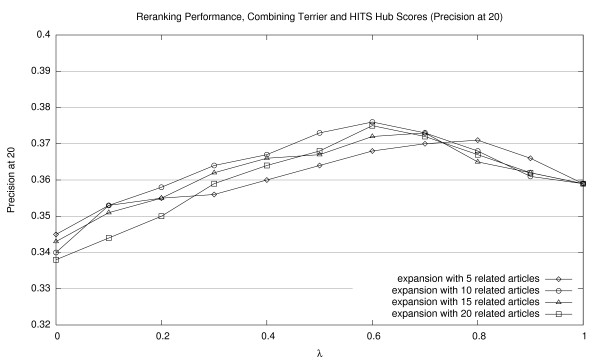
Effectiveness of interpolating Terrier retrieval scores with HITS hub scores (P20).

Corresponding graphs for interpolating Terrier scores with HITS authority scores are shown in Figures 5, 6, and 7 (for MAP20, MAP40, and P20, respectively); and for HITS hub scores, in Figures 8, 9, 10 (for MAP20, MAP40, and P20, respectively). To facilitate comparison between the different methods, we use the same vertical scale for each metric. As with previous figures, we denote statistically significant improvements over the baseline (*p *< 0.05) with solid markers. The only cases observed were with HITS authority scores in P20; in all other cases, gains were not statistically significant. We note the same general trends with both sets of HITS scores, although they appear to be less valuable than PageRank for document ranking. Once again, see Section 5.3 for a discussion of these results.

To provide context, it is worthwhile to compare our results to previous runs submitted to the TREC 2005 genomics track. As Hersh et al. [4] report, the best mean average precision for an automatic run (containing 1000 hits per topic) was 0.289; the best precision at 10 was 0.474 (these were two different runs). The mean for all 58 submitted runs was 0.197 in terms of MAP and 0.358 in terms of P10. As a separate experiment, we generated a comparable baseline run (Terrier using the In_expB2 retrieval model): it achieved 0.255 MAP and 0.428 P10. Since we are using Terrier "out of the box" with minimal modification, we naturally did not expect superlative performance – the best-performing runs all involved techniques to address domain-specific terminology, e.g., through query expansion [6]. Nevertheless, these results confirm that we are starting with a competitive baseline, suggesting that improvements contributed by link analysis are meaningful.

Although a sweep across the *λ *parameter space allows us to understand the relative importance of graph analysis and Terrier retrieval scores, it doesn't tell us if optimal values are realistically achievable. Focusing specifically on PageRank scores (expansion of 20 related articles), we conducted a series of five-fold cross-validation experiments to try and automatically learn *λ *settings (see Section 5.3 for details on the methods). Results are shown in Table 1, along with relative improvements over baseline (original Terrier rankings); all improvements are statistically significant (*p *< 0.05) based on the Wilcoxon signed-rank test. We confirmed that it is possible to obtain optimal effectiveness for a particular metric given appropriate training data.

**Table 1:** Baseline and learned *λ *values for interpolating between Terrier and PageRank scores (expanding 20 related articles).

Tuning on MAP20	
	MAP20

Baseline	0.386
Learned (*λ *= 0.7)	0.416 (+7.8%)*

Tuning on MAP40	

	MAP40

Baseline	0.524
Learned (*λ *= 0.7)	0.544 (+3.8%)*

Tuning on P20	

	P20

Baseline	0.359
Learned (*λ *= 0.6)	0.381 (+6.1%)*

Improvements are statistically significant based on the Wilcoxon signed-rank test (*p *< 0.05).

Our experiments suggest that PageRank is more effective than HITS for analyzing the link structure of related document networks. This makes sense, as the notion of hubs and authorities does not find a natural analog in our application (perhaps the closest is "surveys" and "seminal works"). Whereas HITS assumes a particular linking behavior (which may be true of Web authors), PageRank models a random walk over an arbitrary graph – and appears applicable to both explicit Web hyperlinks and automatically-computed content-similarity links.

## 3 Discussion

### 3.1 Hyperlink Graphs and Related Document Networks

Despite superficial similarities, the analogy between hyperlink graphs of the Web and related document networks in MEDLINE is far from perfect. Hyperlinks are created by humans and represent intentionality. That is, an author links to another Web page because he or she "likes it". Thus, inbound links can be interpreted as votes of confidence with respect to the quality, authority, etc. of the Web page. Algorithms such as PageRank and HITS take advantage of this idea. Related document networks, on the other hand, are artificial. Since they are automatically computed by a content-similarity algorithm, the networks reflect inherent characteristics of the document collection, i.e., term distributions. Furthermore, the nature of content-similarity algorithms means that every document is related to every other one to some degree; thus, we face a thresholding problem when deciding how expansive a related document network might be. Given these important differences, *why *does reranking with PageRank scores improve retrieval effectiveness? Here, we provide independent explanations for our experimental results.

One source of support comes from the cluster hypothesis in information retrieval [7], which is the simple observation dating back several decades that closely associated documents tend to be relevant to the same requests. Another interpretation is that relevant documents tend to occur in clusters. Many researchers have explored and confirmed this hypothesis as a basic property of document collections, albeit to varying degrees [8]. Therefore, the underlying topology of related document networks might provide clues as to where relevant documents might lie in the collection space.

Similar support comes from cognitive psychology. The theory of information foraging [9] hypothesizes that, when feasible, natural information systems evolve toward states that maximize gains of valuable information per unit cost. Furthermore, the theory claims that information seekers behave in a manner that is not unlike our hunter-gatherer ancestors foraging in physical space. One basic assumption in information foraging theory is the notion of information patches – the tendency for relevant information to cluster together. An information seeker's activities are divided between those that involve exploiting the current patch and those that involve searching for the next patch – the user is constantly faced with the decision to pursue one or the other activity. These claims can be understood as a different formulation of the cluster hypothesis: relevant documents co-occur in similarity space, and thus the structure of this space is an important consideration in retrieval. Whereas the cluster hypothesis adopts a system-centered view, information foraging theory focuses on search behavior. Nevertheless, both converge on the same idea.

Additionally, empirical support comes from usage patterns of related article search. A recent analysis of PubMed query logs indicates that searchers click on suggested article titles with significant frequency [10]. Data gathered during a one week period in June 2007 indicate that approximately 5% of page views in non-trivial user sessions (discarding, for example, sessions that consist of a single page view) are generated from users clicking on related article links. Approximately one fifth of all non-trivial user sessions involve at least one click on a related article link. Furthermore, there is evidence of sustained browsing using this feature: the most frequent action following a click on a related article is a click on another related article (about 40% of the time). Thus, related document networks appear to be an integral part of PubMed searchers' activities – suggesting that characteristics of these networks might provide an important source of evidence for document ranking.

### 3.2 Related Work

The related article search feature in PubMed is an instance of "query by example" and can also be understood as a form of single-point relevance feedback. Many commercial search engines provide similar capabilities, through links labeled "similar pages" or "more like this". A number of studies have demonstrated the effectiveness of this feature as a browsing tool [11-13] using simulations of searcher behavior. However, the focus has been on interactive tools for navigating text collections, and not on result ranking.

Cluster-based retrieval has historically received much attention in the information retrieval literature, most recently in the language modeling framework [14]. Clustering can also be used as an interactive search tool, as in Scatter/Gather [15]; cf. [16]. Despite similar goals (exploiting inter-document relationships), clustering represents a different approach from this work. Clustering algorithms typically group together similar documents based on a high-dimensional vector representation. Thus, the relationship of interest is group membership (i.e., a document is a member of the group defined by all documents in the cluster). In contrast, related document networks focus on pairwise content similarity, and require different algorithms for exploiting structure. Diaz's framework of "score regularization" [17], which formalizes the idea that similar documents should have similar retrieval scores, provides an interesting theoretical basis for understanding the relationship between these different classes of techniques.

This work can be viewed as an extension of Kurland and Lee [18], who rerank documents based on generation links induced from language models. They examine application of PageRank and HITS to such graphs, concluding that PageRank outperforms HITS in retrieval experiments. To our knowledge, this article describes the first application of similar techniques to text retrieval in the biomedical domain. Thus, our work generalize the applicability of graph analysis algorithms to a different application area. Furthermore, we relate the effectiveness of this class of techniques to existing browsing features in the PubMed interface and theories of information-seeking behavior, thus establishing a link between interactive retrieval and backend algorithms.

Another interesting use of graph algorithms is seen in the recent work of Lin et al. [19] in the biomedical domain, who apply HITS to a bipartite graph consisting of keywords and documents from MEDLINE. Their goal was to identify important keywords to describe clusters of documents. Although that work is fundamentally different from ours in both aims and methods, it highlights the promise of applying graph algorithms to problems in the biomedical domain – an underexplored approach to an important area.

In addition to information retrieval applications, link analysis has also been adapted for natural language processing tasks. For example, LexPageRank [20] computes PageRank scores over a network defined by sentence cosine similarity, and has been shown to outperform centroid-based techniques for extractive summarization. Other applications of graph-based algorithms in summarization include the work of Mihalcea [21].

## 4 Conclusion

Based on this work, we see a number of future directions worth exploring. Our current approach builds related document networks directly from an initial ranked list – the result is a link graph that is query-biased, i.e., it represents the local neighborhood around a particular region of the document space. We do not know if this is an essential component of our scoring model, or if alternative formulations are equally effective. One might, for example, perform link analysis over the entire document collection (thus generating scores that are query independent). This is likely the preferred approach for operational environments, as it avoids link analysis on the fly (since scores can be pre-computed). Although MEDLINE (currently containing over 17 million records) is relatively small by Web standards, we lack the computational resources and appropriate implementations to perform either PageRank or HITS on the entire document collection. Another interesting possibility is to use related document networks not only for rescoring, but also for expanding the result set. In our reranking experiments, nodes in the related document networks that were not part of the initial result set were used only to define the "local neighborhood" for the PageRank or HITS computation. In principle, however, these nodes might be integrated into results returned to the user.

In summary, we demonstrate that in the absence of explicit hyperlinks, it is possible to exploit networks defined by automatically-generated content-similarity links for text retrieval. Evidence from link structure derived from PageRank scores can be combined with retrieval scores from an off-the-shelf retrieval engine in a straightforward manner. Together, the combination yields significant improvements in ranked-retrieval effectiveness.

## 5 Methods

### 5.1 PageRank and HITS

This work examines two well-known algorithms that exploit link structure to score the importance of nodes in a hyperlink graph such as the Web: PageRank [1] and HITS [2]. We overview both algorithms, but refer the reader to the original articles for details.

PageRank conceptually models a random Web surfer. Given a tireless, idealized user who randomly clicks on hyperlinks (i.e., participates in a random walk), the measure quantifies the fraction of time that the user is expected to spend on any given page. Thus, pages with many in-links or highly-ranked in-links will have high PageRank scores – this is consistent with our intuition of an "important" Web page. The distribution of PageRank scores can be interpreted as the principal eigenvector of the normalized link matrix. As an additional refinement, PageRank incorporates a jump factor, which models the probability that the surfer will randomly jump to another page (thus avoiding link cycles). Typically, PageRank is computed iteratively, and has been empirically shown to converge in surprisingly few iterations, even for extremely large networks.

The HITS algorithm views the hyperlink graph of the Web as containing a set of "authoritative pages" joined together by a set of "hub pages". The task, therefore, is to discover which nodes are hubs and which are authorities from the link structure (i.e., assign a hub and authority score to every node). Operationally, hubs and authorities are recursively defined in terms of each other: a good hub is a page that points to many good authorities, and a good authority is a page that contains links from many good hubs. This gives rise to an iterative technique for computing hub and authority scores, although Kleinberg provides a theoretical foundation for his formulation in terms of eigenvectors of matrices associated with the hyperlink graph.

### 5.2 Test Collection

We evaluated the impact of graph analysis algorithms on retrieval effectiveness with the test collection from the TREC 2005 genomics track. A test collection is a standard laboratory tool for evaluating retrieval systems, consisting of three major components:

• a collection – documents on which retrieval is performed,

• a set of information needs – written statements describing the desired information (called "topics"), which translate into queries to the system, and

• relevance judgments – records specifying the documents that should be retrieved in response to each information need (i.e., which documents are relevant to the topic).

The use of test collections to assess the performance of retrieval algorithms is a well-established methodology in the information retrieval literature, dating back to the Cranfield experiments in the 60's [22]. These tools enable rapid, reproducible experiments in a controlled setting without requiring users. In modern information retrieval research, test collections are created through large-scale evaluations, such as the Text Retrieval Conferences (TRECs) sponsored by the U.S. National Institute of Standards and Technology (NIST) [23].

Experiments in this article were conducted with data from the TREC 2005 genomics track [4]. One salient feature of this TREC evaluation was its use of generic topic templates (GTTs), which consist of semantic types, such as genes and diseases, embedded in prototypical information needs, as determined from interviews with biologists and other researchers. In total, five templates were developed, each with ten fully-instantiated topics; examples are shown in Table 2. In some cases, the actual topics deviate slightly from the template structure (in order to accommodate real requests).

**Table 2:** Templates and sample instantiations from the TREC 2005 genomics track.

#1	**Information describing standard [methods or protocols] for doing some sort of experiment or procedure**.
	*methods or protocols: *fluorogenic 5'-nuclease assay
#2	**Information describing the role(s) of a [gene] involved in a [disease]**.
	*gene: *Transforming growth factor-beta1 (TGF-beta1)
	*disease: *Cerebral Amyloid Angiopathy (CAA)
#3	**Information describing the role of a [gene] in a specific [biological process]**.
	*gene: *APC (adenomatous polyposis coli)
	*biological process: *actin assembly
#4	**Information describing interactions between two or more [genes] in the [function of an organ] or in a [disease]**.
	*genes: *alpha7 nicotinic receptor and ApoE
	*function of an organ: *neurotoxic effects of ethanol
#5	**Information describing one or more [mutations] of a given [gene] and its [biological impact or role]**.
	*gene with mutation: *alpha7 nAChR
	*biological impact: *alcoholism

The TREC 2005 genomics track employed a ten-year subset of MEDLINE (1994–2003) containing 4.6 million citations, or approximately a third of the entire database at the time it was collected in 2004 (commonly known as the MEDLINE04 collection). A total of 32 groups submitted 58 runs to the evaluation. System output was evaluated using the standard pooling methodology for *ad hoc *retrieval, with relevance judgments supplied by an undergraduate student and a Ph.D. researcher in biology. No relevant documents were found for one topic, which was dropped in our experiments.

### 5.3 Statistical Testing and Cross-Validation

When comparing the effectiveness of different retrieval runs, it is of course important to assess the statistical significance of the results. Following established conventions in the information retrieval community, the non-parametric Wilcoxon signed-rank test was used because it makes minimal assumptions about the underlying distribution of differences. Effectiveness metrics (i.e., MAP20, MAP40, and P20) on a per topic basis formed the paired observations.

For the reranking experiments which involved interpolating scores from Terrier and either PageRank or HITS, we compared the effectiveness at each *λ *setting with the baseline Terrier-only run. For each, statistical significance was assessed with a Wilcoxon signed-rank test. However, since each family of tests (i.e., a sweep across the *λ *parameter space) involved multiple comparisons, there is a danger of making Type I errors when considering the entire family of tests. To account for this possibility, we applied the Šidák correction for multiple hypothesis testing [24] – under which we would consider a test significant if its associated probability was smaller than 0.0051 (for a familywise *α *of 0.05). Unfortunately, none of the *p *values associated with the individual Wilcoxon signed-rank tests passed this stringent check (for example, we get *p *= 0.01453 at *λ *= 0.7 in terms of MAP20, expansion with 20 neighbors – corresponding to one of the peaks in Figure 2). Therefore, we cannot claim statistical significance for the entire series of reranking experiments that involved testing multiple *λ *settings. However, it is noted that the Šidák correction is known to be rather conservative; see [25] for discussion.

The complexities associated with multiple hypothesis testing partially motivated our cross-validation runs. Here we provide more details about the experimental procedure for results presented in Section 2.2. We conducted three separate five-fold cross-validation runs, tuning on each metric in turn. Topics were stratified in such a way that each fold contained a proportional representation of each template. We trained on four folds and tested on the fifth (selecting the *λ *that maximized the metric in question from the training topics). That is, for each fold we trained on eight topics from each template and tested on the remaining two topics for that template. This process was repeated five times, with a different set of training and held-out test topics each time. Although topics varied in terms of difficulty and in terms of the number of relevant documents, they represented the most natural unit for sampling since each topic corresponds to an information need. The stratification procedure across templates ensured that each fold contained a balanced representation of each *class *of information need.

Elaborating on the results in Section 2.2, details of our cross-validation experiments with PageRank are presented in Table 3. We show three separate runs, tuning on MAP20, MAP40, and P20. For each metric, the effectiveness on the training set and on the held-out test set for each fold is presented. Each cell in the table contains the mean followed by the standard deviation. For reference, in the final column we show the baseline effectiveness (i.e., Terrier-only scores) on the same held-out test topics. In terms of all three metrics, we see consistent improvement over the baseline for all folds. These results suggest that the improvements achieved by combining PageRank and Terrier scores are indeed statistically significant.

**Table 3:** Detailed results for five-fold cross-validation experiments, interpolating PageRank and Terrier scores (expansion of 20 related articles).

Tuning on MAP20			
Fold	training (*λ *= 0.7)	testing (*λ *= 0.7)	baseline

1	0.390 ± 0.275	0.514 ± 0.298	0.461 ± 0.304
2	0.447 ± 0.281	0.294 ± 0.260	0.264 ± 0.233
3	0.403 ± 0.273	0.473 ± 0.328	0.465 ± 0.313
4	0.439 ± 0.292	0.325 ± 0.225	0.277 ± 0.203
5	0.400 ± 0.284	0.477 ± 0.276	0.472 ± 0.300

Tuning on MAP40			

Fold	training (*λ *= 0.7)	testing (*λ *= 0.7)	baseline

1	0.513 ± 0.346	0.668 ± 0.344	0.636 ± 0.338
2	0.590 ± 0.343	0.365 ± 0.322	0.338 ± 0.290
3	0.539 ± 0.350	0.567 ± 0.356	0.560 ± 0.349
4	0.557 ± 0.343	0.495 ± 0.382	0.461 ± 0.382
5	0.400 ± 0.284	0.629 ± 0.317	0.627 ± 0.327

Tuning on P20			

Fold	training (*λ *= 0.6)	testing (*λ *= 0.6)	baseline

1	0.355 ± 0.330	0.520 ± 0.375	0.475 ± 0.366
2	0.421 ± 0.353	0.265 ± 0.277	0.250 ± 0.247
3	0.411 ± 0.349	0.289 ± 0.310	0.272 ± 0.299
4	0.379 ± 0.330	0.425 ± 0.404	0.400 ± 0.406
5	0.377 ± 0.348	0.435 ± 0.329	0.425 ± 0.338

Mean and standard deviation for each fold are shown; for reference, baseline results on the test topics are provided. Note that optimized effectiveness metrics show consistent improvements over baseline.
